# Supplementary material for: Rhizosphere Microbiota Promotes the Growth of Soybeans in a Saline–Alkali Environment under Plastic Film Mulching
Source: Plants (Basel). 2023 May 5;12(9):1889. doi: 10.3390/plants12091889 (PMC10180738; doi:10.3390/plants12091889)
Supplement: Supplementary file 1 [file plants-12-01889-s001.zip › plants-2361249-supplementary.pdf]

Rhizosphere Microbiota Promotes the Growth of Soybeans in a Saline–Alkali Environment under Plastic Film Mulching

Han-Cheng Mao, Yifei Sun, Chengyuan Tao, Xuhui Deng, Xu Xu, Zhenquan Shen, Laijie Zhang, Zehui Zheng, Yanhua Huang, Yongren Hao, Guoan Zhou, Shulin Liu, Rong Li, Kai Guo, Zhixi Tian and Qirong Shen

Supplementary Files:

Table S1. Twelve soybean cultivars and field yield information in this study.

	Cultivar names	Treatments	
		C	CF
Field yield ($g \cdot plant^{-1}$)	wlms82	7.93	14.39
	Hedou13	10.98	21.68
	TZX-805	15.64	23.27
	TZX-1736	12.11	19.97
	TZX-3288	8.63	18.34
	TZX-3736	11.13	22.09
	TZX-3763	6.43	9.94
	TZX-3782	9.02	14.39
	TZX-3873	5.10	7.83
	TZX-773	4.27	8.65
	TZX-3376	6.33	9.29
	TZX-3766	6.04	14.11

Note: C = chemical fertilizer; CF = chemical fertilizer with plastic film mulching.

Table S2. Characteristics of bulk (Dongying) and experimental (Yancheng) soil.

Targets	Unit	Bulk soil		Experimental soil
		C	CF	-
Moisture content	%	13.60 ±0.15	14.36±0.09	8.26±0.05
Electrical conductivity	$\mu s \cdot cm^{-1}$	602.31±36.88	437.19±11.41	506.93±4.93
K^+	$g \cdot kg^{-1}$	0.05±0.00	0.04±0.00	0.09±0.00
Na^+	$g \cdot kg^{-1}$	0.72±0.02	0.48±0.01	0.68±0.00

Note: C = chemical fertilizer; CF = chemical fertilizer with plastic film mulching. Values are means ± standard errors. Methods: moisture content was calculated as dry soil weight (soil samples were placed at 105 °C for 12 h) divided by fresh soil weight; Electrical conductivity (soil/water ratio: 1:10) was determined by using an EC meter (FE28, METTLER TOLEDO, Shanghai, China); K Ions and Na Ions were determined by using flame photometry.

Table S3. PERMANOVA results of bacterial and fungal community structures at the OTU level.

Soil compartment	† Main test	Bacteria			Fungi		
		PERMANOVA			PERMANOVA		
		F	R^2	*P* (>F)	F	R^2	*P* (>F)
Rhizosphere soil	treatments	8.58	0.08	< 0.01	9.24	0.09	< 0.01

Note: PERMANOVA, permutational multivariate analysis of variance. † Main test represents treatments C (chemical fertilizer) and CF (chemical fertilizer with plastic film mulching). The values in the table represent the pseudo-F ratio (F), the estimation of the variance component (R^2) and the level of significance for PERMANOVA.

Table S4. Two-way ANOVA results of the agronomic characteristics of soybean in the greenhouse experiment.

Agronomic characteristics	† Main test	Two-way ANOVA	
		F	*P* (>F)
Aboveground fresh biomass	treatment 1	5.63	0.028 (*)
	treatment 2	34.01	1.29E-05 (***)
	treatment 1 × treatment 2	3.38	0.08 (NS)

Note: Two-way ANOVA, two-way analysis of variance. † Main tests represent treatment 1 (S: sterilized saline-alkali soil, US: unsterilized saline-alkali soil) and treatment 2 (NF: without plastic film mulching, F: with plastic film mulching). Values in the table represent the pseudo-F ratio (F) and the level of significance for two-way ANOVA.

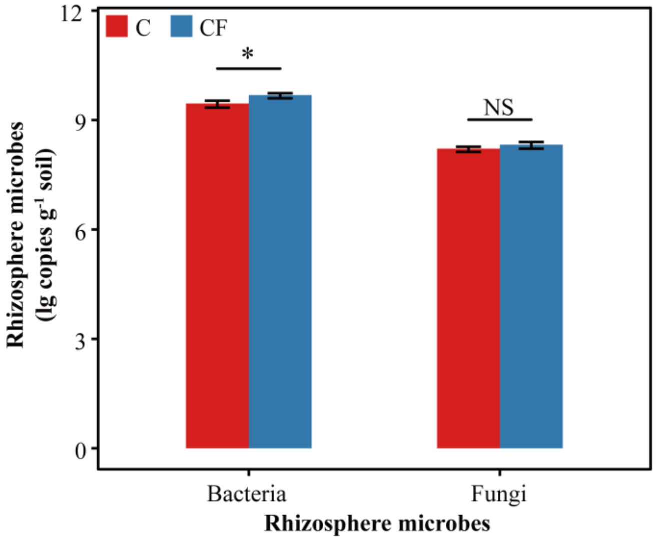

Figure S1. Abundance of total rhizosphere bacteria and fungi based on qPCR.

C = chemical fertilizer, CF = chemical fertilizer with plastic film mulching. qPCR, quantitative polymerase chain reaction. Asterisks above the bars indicate significant differences between the two treatments as determined by Student's t test using 0.05 as the boundary (* = $P < 0.05$, NS = not significant). Values = mean ± standard error.

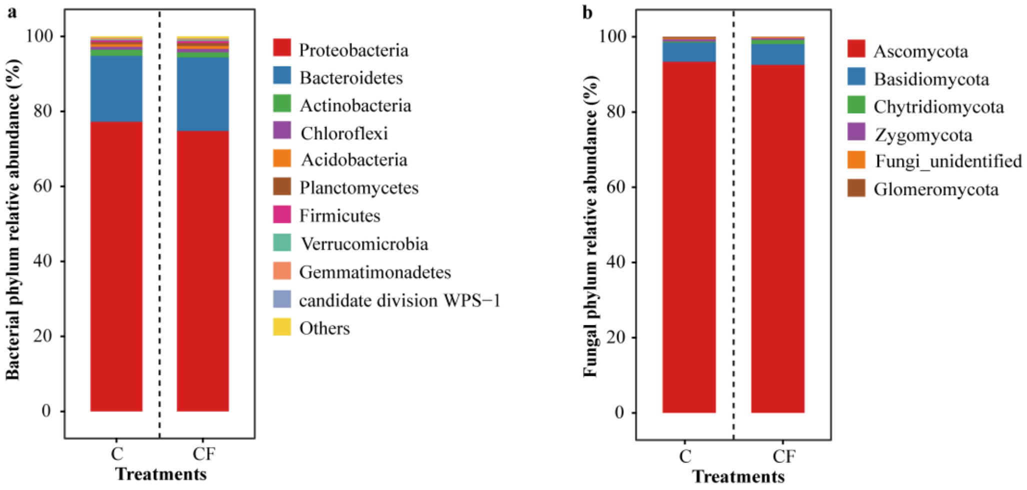

Figure S2. Relative abundances of bacteria (a) and fungi (b) at the phylum level.

C = chemical fertilizer, CF = chemical fertilizer with plastic film mulching.

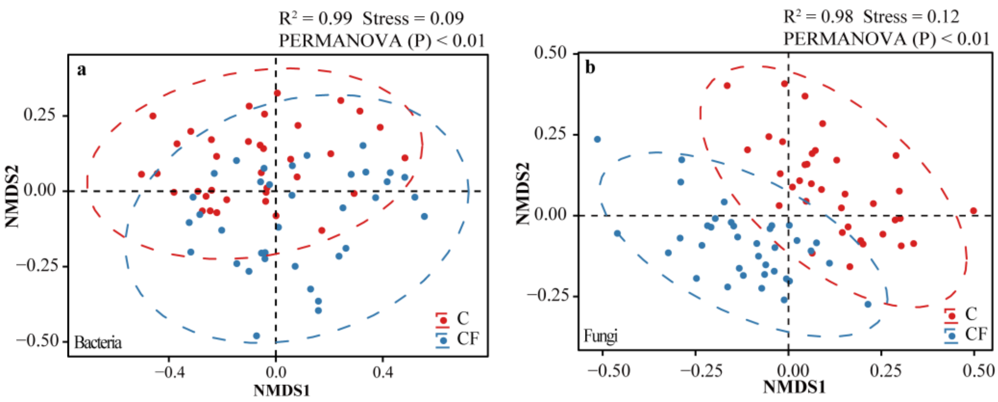

Figure S3. Nonmetric multidimensional scaling (NMDS) of bacterial (a) and fungal (b) community structures based on Bray-Curtis distance.

C = chemical fertilizer, CF = chemical fertilizer with plastic film mulching. PERMANOVA, permutational multivariate analysis of variance. Ellipses cover 95% of the data for each treatment.

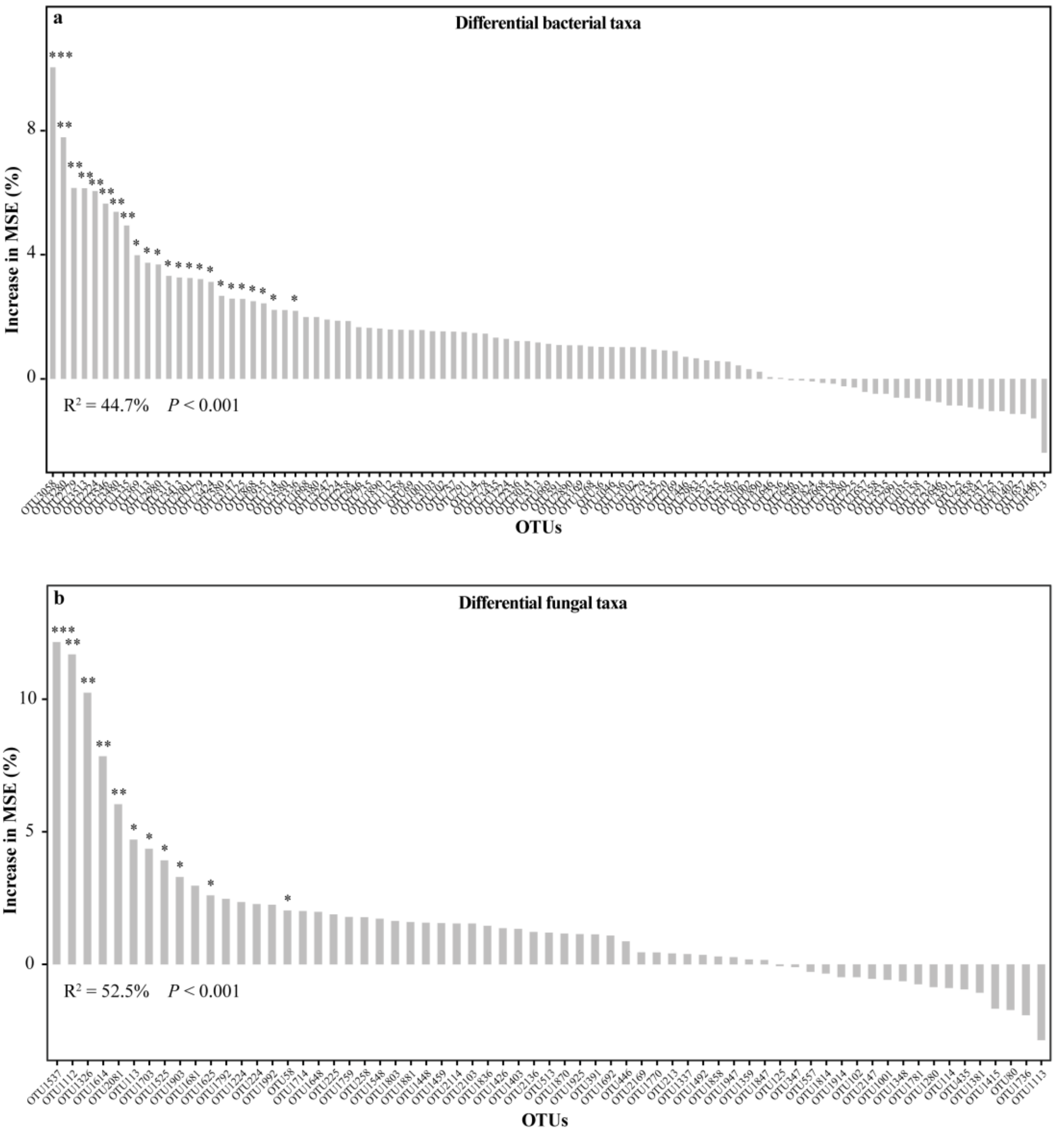

Figure S4. Random forest model of influences of differential bacterial (a) and fungal taxa (b) on soybean yield.

OTUs, operational taxonomic units. Percentage increases in the MSE (mean squared error) of variables represent the importance of predictors. *** = $P < 0.001$, ** = $P < 0.01$, * = $P < 0.05$.
